# Iron Chelation Therapy in Thalassemia Syndromes

**DOI:** 10.4084/MJHID.2009.034

**Published:** 2009-12-29

**Authors:** Paolo Cianciulli

**Affiliations:** Day Hospital Talassemia, Ospedale S. Eugenio, Roma

## Abstract

Transfusional hemosiderosis is a frequent complication in patients with transfusion dependent chronic diseases such as thalassemias and severe type of sickle cell diseases. As there are no physiological mechanisms to excrete the iron contained in transfused red cells (1 unit of blood contains approximately 200 mg of iron) the excess of iron is stored in various organs. Cardiomyopathy is the most severe complication covering more than 70% of the causes of death of thalassemic patients. Although the current reference standard iron chelator deferoxamine (DFO) has been used clinically for over four decades, its effectiveness is limited by a demanding therapeutic regimen that leads to poor compliance. Despite poor compliance, because of the inconvenience of subcutaneous infusion, DFO improved considerably the survival and quality of life of patients with thalassemia. Deferiprone since 1998 and Deferasirox since 2005 were licensed for clinical use. The oral chelators have a better compliance because of oral use, a comparable efficacy to DFO in iron excretion and probably a better penetration to myocardial cells. Considerable increase in iron excretion was documented with combination therapy of DFO and Deferiprone. The proper use of the three chelators will improve the prevention and treatment of iron overload, it will reduce complications, and improve survival and quality of life of transfused patients.

## Introduction:

Iron overload occurs when the intake of iron is increased over a prolonged period of time and is commonly seen in patients with hereditary or refractory anemias (e.g. β-thalassaemia major, sickle cell anemia and myelodysplastic syndromes) who receive frequent blood transfusions. The iron excess is initially stored in the reticuloendotelial system, which has a capacity of about 10–15 g, and then in all parechymas,^1^ resulting in life-threatening complications, namely cardiopathy, liver and endocrine dysfunction and reduced patient’s survival.^2^,^3^,^4^ Iron excess also increases cell concentration of iron-binding proteins such as ferritin and haemosiderin complexes in lysosomes^5^. Non-transferrin-bound-iron (NTBI), iron in low molecular weight forms, may initiate free radicals reactions.^6^ Patients with β-thalassaemia require regular blood transfusion in order to have a normal life. Correct management inhibits bone marrow hyperactivity and delays the appearance of hypersplenism. Their mean annual intake is 165 or 140 mg of pure red cells/Kg (for non splenectomized and splenectomized patients, respectively), which corresponds to 0,49-0,44 mg/Kg/day.^7^ Prior to the introduction of chelating therapy, most patients did not reach the second decade of life, mainly owing to heart disease.^8^

## Desferrioxamine:

The hexadentate chelator desferrioxamine B (DFO) was identified as the first effective biologically active Fe chelator. Released in the 1960s as the first clinically approved chelator for the treatment of iron overload, DFO has significantly improved the life expectancy and the quality of life of patients with iron overload^9^ who previously would not have survived after their teen years. Moreover, it had been the gold standard in iron chelation therapy. The ability of a chelating agent to penetrate cells depends on its molecular size and affinity for lipids. For its large molecular size (molecular weight 656 Daltons) and low affinity for lipid, DFO is poorly absorbed from the gastrointestinal tract.^10^ It is not orally active, undergoes rapid renal elimination. Thus, it must be administrated only parenterally. Its plasma half-life is short (∼20 minutes). It binds iron very strongly with a ratio of 1:1. The usual dose of DFO is 20 to 60 mg/Kg/die. It is subcutaneously administered via a battery-operated portable pump over a period of 8–12 hours overnight, for 5 to 7 nights per week. This has resulted in a large proportion of patients (∼ 33%) failing to comply with this regimen.^11^

## Deferiprone:

Deferiprone is a synthetic, bidentate iron chelator. Three molecules of deferiprone are required to bind one atom of iron. Deferiprone also binds other metals including zinc; zinc deficiency has been reported in a small number of patients with iron overload receiving long-term deferiprone.^12^ It is orally administered at 75 mg/Kg/day in three divided doses. Compliance rates with deferiprone are generally higher than those associated with subcutaneous infusions of desferrioxamine.^13^ The molecule undergoes extensive liver metabolism and > 85% of the administered dose is recovered in the urine as a non-chelating O-glicuronide conjugate. It is excreted predominantly via the renal system as the parent compound, its conjugate and as an iron-bound complex. The urinary iron excretion obtained with a dose of 75 mg/Kg is comparable with that obtained with 40–50 mg/Kg DFO infusion, while iron excretion in the stool is negligible. Deferiprone was introduced in Europe in 2000 as second-line therapy for β-thalassemia patients with DFO–related adverse events or contraindications to DFO. In general, the above indicated oral daily dose is required for the treatment of iron overload condition, although some investigators have used up to 100 mg/Kg/day. The most frequent deferiprone-related adverse reactions are gastrointestinal adverse reactions (nausea, abdominal pain, vomiting) and arthralgia, while the most serious adverse event is agranulocytosis.^12^ In a multi-centre study designed to evaluate the incidence of agranulocytosis (neutrophil count of < 0.5 x 10^9^/L) in patients treated with deferiprone, 1 patient out of 187 (0,5%) was affected by this condition and 9 patients (4,5%) had moderate neutropenia (two consecutive neutrophil counts of 1.5 x 10 ^9^/L) within the first year of treatment.^14^ Over the following three years, there were no cases of agranulocytosis observed and seven new cases of moderate neutropenia were reported.^15^ In an Italian study conducted in 532 patients with β-thalassemia treated for a total of 1154 patients-years, the rates of agranulocytosis and neutropenia were 0.43 and 2.08 respectively, per 100 patients-years.^16^ An idiosyncrasic pathogenesis can be hypothesized.^17^ Generally it is more frequent during the first year of deferiprone treatment. Agranulocitosys is sometimes reversible when the drug is stopped but sometimes can be necessary to quickly begin therapy with GCS-F. For elevated incidence of relapse, it is not suitable to submit the patient to the same therapy. An interesting advantage of deferiprone over DFO seem to be its ability to reduce cardiac iron levels in patients with β-thalassemia, wich is probably due to the small size of the molecule (molecular weight 139 versus 656 Dalton for DFO) and to its lipophilic properties. Anderson et al. 14,15,18 demonstrated that patients with β-thalassemia treated with deferiprone (n=15) presented a significantly (p=0.02) elevated myocardial T2* value, an RMI variable with inverse correlation to tissue iron levels, compared with that of mached control patients (n=30) treated with DFO, and that more than half (67%) of the patients treated with DFO were not protected against cardiac siderosis. In contrast, almost three quartes (73%) of deferiprone recipients were protected against cardiac siderosis.^19^ Furthermore in such patients who were randomized to open-label deferiprone at mean dose of 92 mg/Kg (n=29) or who continued to receive subcutaneous DFO at standard dose (n=32), improvements from baseline in myocardial T2* values at 12 months (primary endpoint) were significant for both groups (p< 0.001 for both groups), but the between-group difference was significantly in favor to deferiprone (27% versus 13% improvement; p< 0.0023). In the same study, in patients treated with deferiprone, left ventricular ejection fraction improved by 3.1% (absolute units), compared with 0.32% improvement in DFO-treated patients (p=0.003 versus DFO).^20^ Recently, some authors in a multicenter, prospective, long-term (7 years and 4 months) randomized trial on 265 enrolled thalassemic patients, have shown an improving of survival in patients with deferiprone treatment (alone or in sequential or in association with DFO) versus DFO.^21^ Recently El-Beshlawy et al. in a multi-centre 24 week study period on 100 young thalassemic patients ≤ 10 years old showed that the a new liquid formulation had a lower incidence of gastrointestinal adverse reactions that previously reported with the tablet formulation (13% vs 42%), no important changes about neutropenia or agranulocytosis were observed.^22^ Hoffbrand’s metanalysis^18^ demonstrated that treatment with deferiprone leads to a negative iron balance in some but not in all patients. Factors that may contribute to inter-individual response variation are the degree of iron overload, therapy duration, dosage, and compliance. One explanation for individual variation in response to the drug is that deferiprone is inactivated by glucuronidation, which may be very rapid in some individual.^12^

## Combined therapy:

Deferiprone can be associated to DFO when the chelation response is unsatisfactory or under conditions of serious cardiac siderosis with or without heart failure, in conditions with high risk of morbility or mortality. The efficacy of the combination of a low molecular weight chelating agent that is able to penetrate cells efficiently, with a high molecular weight chelating agent that is able to form a stable association with iron and thus achieve a satisfactory urinary iron excretion, has been shown in several clinical studies.^23^–^25^ Combination therapy leads a reduction of plasma ferritin levels in patients in which monotherapy with deferiprone failed to produce a satisfactory outcome^26^ and shows an additive effect on the urinary excretion of iron.^27^ Tanner et al demonstrated that, compared with DFO monotherapy, combination therapy significantly improved myocardial T2* values (primary endpoint), plasma ferritin levels, LVEF, endothelial function.^23^ This approach has also proven effective in the acute phase treatment of heart failure caused by iron overload.^29^,^30^ Recently, the beneficial effect of combination therapy on the pancreatic endocrine damage secondary to hemosiderosis has been described.^30^ The mortality due to cardiac damage is strongly reduced by means of T2* cardiovascular magnetic resonance and combined therapy with deferiprone and desferrioxamine.^31^

## Deferasirox:

Deferasirox (ICL670) is a new iron chelator orally bioavailable. Well as Thalassemia Major and Intermedia, it has been used in many others chronic anemias transfusions dependent such as Sickle Cell Disease (SCD), Diamond Blackfan anemia, Myelodysplastic Syndromes (MDS) and other rare anemias. In 2005, Food and Drug Administation (FDA),^32^ and afterward the European Medicine Agency (EMEA)^33^ approved its use in Thalassemia Major patients (aged ≥ 6 years), transfused with ≥ 7ml/kg/month of packed red blood cells. Deferasirox therapy is allowed also in the following groups of multitransfused iron overloaded patients when treatment with DFO is contraindicated or inadeguate: Thalassemia Major patients transfused with <7ml/kg/month of packed red blood cells, Thalassemia Major patients aged 2–5 years, patients affected by other anemias. Actually, Deferasirox is available in 90 nations.^34^

Deferasirox, a N-substituted bis-hydroxyphenyltriazoles compound, belongs to a new class of tridentate iron chelators. It was selected among more than 700 molecules because it is orally administrable and it gave the best therapeutic results.^34^,^35^ Two molecules of Deferasirox are able to generate a stable complex with one molecule of iron. Its half life (t1/2) is between 8 and 16 hours, allowing a once-daily administration. After the oral administration (dissolved in water or orange or apple juice) it is rapidly absorbed and its plasma level is in the therapeutic range for 18 to 24 hours^36^. Thus, after a dose, the chelating effect lasts all day. The lowest plasma level of the drug, during the day, corresponds to 25% of the peak of drug plasma concentration.

Deferasirox protects cells from the toxicity of Non Transferrin-Bound serum Iron (NTBI) and of Labile Plasma Iron (LPI). The last generates Reactive Oxigen Species (ROS) able to damage cells^37^. High LPI values, if they frequently occur, can affect the most important organ function and the patient survival.^38^ Also the other chelators (DFO and Deferiprone) have this protective action but, for their shorter half-life, this is not continuous. On the contrary, Deferasirox assures a more constant chelation during the day. Daar et al demonstrated that Deferasirox, after two hours of the oral intake in the morning, reduces LPI levels. During the treatment, if the drug intake is regular, the LPI, measured before the daily dose, tends to decrease and subsequently LPI values reach the normal range.^30^,^40^

If Deferasirox is taken with food, its bioavaibility changes overall according to the fat content of the meal. For this reason the drug must be taken at least 30 minute before eating, preferably at the same time every day.^41^ The patient age is another parameter influencing the bioavailability which is lower for the adolescents (12–17 years old) and children (2–12 years old).

Metabolism and elimination of deferasirox and the iron chelate (Fe-[deferasirox]2) is primarily by glucuronidation followed by hepatobiliary excretion into the feces (83%). A enterohepatic circulation, after a decojugation of glucuronidated drug in small bowel, is supposed.

Many clinical trials, performed in patient groups with transfusion-dependent anemias, demonstrated Deferasirox efficacy.^36^,^43^,^46^ For this purpose serum ferritin and Liver Iron Concentration (LIC) were monitored. These study showed that a relation between the drug dose administered and the reduction of body iron index is present and this is a function of iron intake though transfusions.^47^,^48^ In fact, treating, multitransfused β-thalassemia patients with 20 and 30mg/kg/die, a mean LIC reduction ranging from −0.4 and −8.9 mg Fe/g of dry weight and a mean ferritin reduction ranging from −36 and −926 μg/l were observed. These result obtained after an year were confirmed after a follow-up of 5 years-Moreover, during this time, the number of patients who reached a LIC<7 mg Fe/g dw and serum ferritin < 1000 μg/l progressively raised (35% versus 45% for LIC and 12% versus 33% for ferritin).^49^

Analysis of registration study results, indicated that the Deferasirox dose must be set in function of transfusion regimen and in function of the therapeutic target: reduction of iron overload or maintenance of iron balance. The daily dose of 20 mg/kg/was able to obtain a neutre/negative iron balance in 47% of patients with high blood supply, in 55% of patients with intermediate blood supply and in 75% of patients with low blood supply. The daily dose of 30 mg/kg was sufficient to reduce iron overload in 82% of patients with high blood supply, in 83% of patients with intermediate blood supply and in 96% of patients with low blood supply. Thereafter 30 mg/kg/die is the starting dose to reduced iron overload in regularly highly multitransfused patients.^50^

Recently EPIC study results were published.^51^ This study was performed examining 1744 multitransfused overloaded patients: 1115 β thalassemic (63.9%); 341 with MDS (19.6%); 116 with Aplastic Anemia (6.7%); 80 SCD (4.6%); 43 with rare anemias (2.5%) and other anemias including cancer anemias (2.9%). For a mean Deferasirox dose of 22,2 mg/kg/die and an iron intake of 0.41 mg Fe/kg/die, the mean serum ferritin reduction was 264 ng/ml. In a cohort of patients treated with 30 mg/kg/die or more the highest reduction of ferritin was registered (-882 ng/ml with an iron intake of 0.37 mg Fe/kg/die).

The ESCALATOR study^52^ included 237 patients with high overload and not responders to other chelators. They were treated with Deferasirox dose > 30mg/kg/die. The mean basal LIC was 20.1 mg Fe/g dw; at the end of the study (after 2,7 years) the mean LIC was significantly lower: 11.8 mg Fe/g dw (*P*<0.0001).^53^ The cardiac T2* was evaluated in 19 patients of the ESCALATOR study: this parameter showed a significant improvement, independently to the mean basal values.^54^

Moreover, in multitransfused patients Deferasirox is efficacious in reducing cardiac overload and in preventing it.^55^–^57^ In fact, in 100 β thalassemic, in a period of 2 years, an improvement of T2* of 40,8% was observed (mean T2* 11.2 msec versus 12,9 msec p<0.0001). In 78 β thalassemic with normal basal T2* after a year no change was observed. Finally in those patients showing a basal T2* ranging between 10 and 20 msec, a mean dose of 34.5±4.8 mg/kg/die for two years normalized the T2*: Δ% = +48.1%; basal T2* 14.6 msec versus final T2* 20,4 msec; p<0.001.

Moreover Deferasirox is a safe and well tolerated drug in adults and children both in short and long treatment periods. In registration studies after 5 years, no new side effects were reported and no change in the incidence of those already reported. The frequency of the most common side effects has been progressively decreasing.^36^, ^43^–^46^,^51^–^57^

Table 1 summarized the most important reported side effects. At present, this drug is largely employed. Among the side effects not reported in registration studies, cytopenias occurred, although mainly in patients with predisposing clinical conditions. The exact role of Deferasirox in inducing cytopenias is not defined. Nevertheless, regular controls of complete and differential blood counts are recommended. Renal and liver failures and bleeding from the gastroenteric tract were reported. Similarly to cytopenias, in the clinical history of these patients, predisposing conditions able to induce these complications are often recognizable. Before starting Deferasirox therapy, the careful patient physical examination, the accurate medical history registration and the detection of the most common laboratory tests are crucial for a safe use of the drug.^58^ The results of many clinical trials and studies are consistent with the evidence that Deferasirox is efficacious in removing and in preventing the liver and cardiac iron overload, in chelating free iron for 24 hours and it is safe and well tolerated also if high doses (up to 40 mg/kg/die) are used.

Table 2 show the most important feature of iron chelators commercially available

## Conclusions:

The recent expansion of erythrocyte transfusional therapy to pediatric patients with sickle cell disease and an elevated risk of stroke^59^ and in some type of thalassemia intermedia associated with bone deformations and growth defects, has increased the population of pediatric patients requiring chelation therapy. The knowledge of the properties of the available iron chelating agents and of individual patient requirements, coupled with effective methods to accurately monitor iron levels, has enabled iron chelation therapy to be highly personalized. The recently available oral chelators offer a convenient administration regimen, overcoming the burden of a subcutaneous infusions of DFO and potentially improving compliance. Treating patients with tailored therapy will eventually lead to improvement in morbidity and mortality induced by iron toxicity.

## Figures and Tables

**Table 1.** Side effect overview.

**Side effects**	**Frequence (Patient %)**	**Notes**
Abrupt creatinine elevation	36	Mild; generally it remains within the normal range; often spontaneously regresses; the reduction of the dose can normalize creatinine level
Gastroenteric disorders (nausea, vomit, diarrhea, abdominal pain)	26	Dose-dependent; mild-moderate; generally they regress
Rash	7	Dose-dependent; mild-moderate; generally they regress
Liver enzyme elevation	2	Not dose dependent: Often liver enzymes were high before starting the therapy. Elevation> 10 UI are not common (0.3%)
Reduction of hearing Cataract	≤1	Not common

**Table 2.** Characteristics of Cheletors at present available.

**Charatteristics**	**Deferoxamine**	**Deferiprone**	**Deferasirox**
Dose (mg/Kg/day)	25–60	75	20–30
Route of administration	sc, ev (8–12 hours, 5days/week)	Orally, three times/day	Orally, once a day
Half life	20–30 min	3–4 hours	8–18 hours
Excretion	Urine, stools	Urine	Stools
Most important side effects	Local skin reactions, allergies, neuro-sensorial damage, growth retardation	Gastroenteric disorders, agranulocytosis / neutropenia arthralgias, liver enzyme elevation	Gastroenteric disorders, rush, mild not progressive creatinine elevation, liver enzyme elevation
Status	Approved	Approved except in USA/Canada	Approved
